# Expression of p16 and p21 in the frontal association cortex of ALS/MND brains suggests neuronal cell cycle dysregulation and astrocyte senescence in early stages of the disease

**DOI:** 10.1111/nan.12559

**Published:** 2019-06-17

**Authors:** I. Vazquez‐Villaseñor, C. J. Garwood, P. R. Heath, J. E. Simpson, P. G. Ince, S. B. Wharton

**Affiliations:** ^1^ Sheffield Institute for Translational Neuroscience University of Sheffield Sheffield UK

**Keywords:** amyotrophic lateral sclerosis, cell cycle dysregulation, DNA damage, DNA damage response, motor neurone disease, oxidative stress, senescence

## Abstract

**Aims:**

Cellular senescence plays a role in organismal ageing and has been linked to persistent DNA damage in age‐related diseases. Brain senescence has been described in astrocytes and microglia, but it is less well understood in neurones. Evidence suggests that neurones activate a senescence‐like mechanism that could contribute to neurodegeneration. We aimed to determine whether a persistent DNA damage response (DDR) and senescence activation are features of motor neurone disease (amyotrophic lateral sclerosis, ALS/MND).

**Methods:**

We examined expression of senescence (p16 and p21) and DNA damage markers (8‐OHdG and γH2AX) in motor cortex (MCx), frontal association cortex (FACx) and occipital cortex (OCx) in post‐mortem tissue donated by patients with ALS/MND and controls.

**Results:**

Nuclear expression of p16 and p21 was detected in glial cells; double immunofluorescence for p16/p21 and glial fibrillary acidic protein (GFAP) suggested that some of these cells were GFAP ^+^ astrocytes. p21 nuclear expression was also found in neurones. Higher levels of p16^+^ (glia, *P = 0.028*) and p21^+^ (glia, *P = 0.003*; neurones, *P = 0.008*) cells were found in the FACx of ALS/MND donors but not in the MCx or OCx. Expression of p16 and p21 did not correlate with 8‐OHdG or γH2AX.

**Conclusions:**

Expression of p16 and p21 in glia, mainly in astrocytes, suggests senescence induction in these cells; however, neuronal p21 expression might reflect a more general mechanism of age‐related cell cycle dysregulation. The significantly higher proportion of cells expressing either p16 or p21 in the FACx of ALS/MND donors could indicate senescence activation and cell cycle dysregulation in early stages of the disease.

## Introduction

Cellular senescence is the loss of replicative capacity in mitotic cells that occurs in response to different stimuli, including oncogene activation, oxidative stress and DNA damage 1, 2, 3, 4. Activation of senescence is modulated by two main pathways: the p16 and p21 tumour suppressor signalling cascades, which promote an irreversible cell cycle arrest and cause changes in cell morphology, chromatin organization and in mitochondrial and lysosomal function 2, 5, 6, 7. Senescent cells also show changes at the gene expression level reflected in an altered secretory profile, known as the senescence‐associated secretory phenotype (SASP). The SASP involves the secretion of pro‐inflammatory cytokines, chemokines, growth factors and proteases that can act in an autocrine and paracrine manner to reinforce senescence and to spread this phenotype to surrounding cells 8, 9, 10. Although accumulation of senescent cells is implicated in normal ageing, it has been shown that this accumulation can become detrimental, promoting degeneration and loss of tissue function, thus contributing to age‐related diseases 11, 12.

Senescence in the ageing brain has been described in astrocytes and microglia, suggesting that this mechanism could contribute to the disruption of the glia–neuron interaction through the SASP and to the development of age‐related brain pathologies 13, 14, 15, 16, 17. Induction of senescence in post‐mitotic cells such as neurones is less well understood, but evidence of senescence and a SASP in post‐mitotic neurones of ageing mice suggests that this mechanism might not be restricted to proliferating cells or to *in vitro* conditions. In this study, the expression of p21 and IL‐6 production, one of the SASP components, correlated with DNA damage accumulation in Purkinje neurones of ageing mice. Moreover, the frequency of senescent‐like neurones increased with the age of the mice, linking this mechanism in neurones to ageing *in vivo* 18. Markers of a neuronal DNA damage response (DDR) and of senescence in an ageing human cohort have also been found to correlate with cognitive impairment at the early stages of Alzheimer's disease (AD) 19, suggesting that DNA damage and activation of a DDR in the ageing human brain could contribute to neurodegeneration through the activation of senescent pathways. Based on this evidence, induction of senescence in the brain would not be restricted to glial cells, but could also occur in neurones, leading to the development of a senescent‐like neuronal phenotype, similar to the SASP, that could accelerate neuronal dysfunction and promote the development of neurodegenerative diseases, such as AD, Parkinson's disease (PD) and amyotrophic lateral sclerosis (motor neurone disease; ALS/MND).

We have investigated the expression of senescence markers in the brain of patients with ALS/MND, an age‐related disorder characterized by motor neurone degeneration in the motor cortex, brainstem and spinal cord, resulting in progressive muscle weakness eventually leading to death 20, 21. Increasing evidence supports a role for oxidative DNA damage 22 and impaired DNA repair mechanisms in response to oxidative stress in the pathogenesis of ALS/MND 23. In the current study, we used an immunohistological approach to determine whether oxidative DNA damage and the activation of a DDR in ALS/MND associated with activation of senescence pathways in the brain, specifically determining neuronal and glial expression of cellular senescence‐associated proteins (p16 and p21), oxidative DNA damage (8‐OHdG) and a marker of DDR activation (γH2AX) in post‐mortem brain tissue of ALS/MND and control donors.

## Methods

### Cohort

Formalin‐fixed paraffin‐embedded (FFPE) and frozen tissue samples of motor cortex (MCx), frontal association cortex (FACx) (Brodmann area 8/9) and occipital cortex (OCx) from ALS/MND and control donors were obtained from the Sheffield Brain Tissue Bank (SBTB). Formalin‐fixed paraffin‐embedded blocks from the three regions were available for 10 ALS/MND cases and for nine controls. Frozen blocks from the MCx and FACx were available for eight ALS/MND patients and five MCx and four FACx controls. The cohort consisted of 16 male and seven female control donors (one not recorded for sex, two not recorded for age, mean age of 68.95 years, range 26–84) and six male and four female ALS/MND donors (mean age of 64.33 years, range 48–84). The mean post‐mortem delay (PMD) for controls was 27.66 h (range 5–75 h) and for the ALS/MND group was 35.62 h (range 9–96 h). No record of PMD was available for two ALS/MND donors and for seven controls. One of the donors included in this study was diagnosed with frontotemporal dementia (FTD)‐ALS/MND. Detailed information about the ALS/MND and control donors is shown in Table 1.

**Table 1:** Age, sex, post‐mortem delay (PMD) and clinical diagnosis of the donors that compose the MND/control cohort

Group	Case	Age (y)	Sex	Diagnosis	C9orf72	PMD (hrs)	Region	Type of tissue
ALS/MND	1	48	M	Familial—PD	+	23	MCx and FACx	FFPE and frozen
	2	79	M	Familial	+	13		
	3	64	M	Sporadic	+	Not rec.		
	4	59	F	Familial—FTD	+	28		
	5	51	F	Sporadic	−	40		
	6	Not rec.	M	Sporadic	−	9		
	7	66	M	Sporadic	−	96		
	8	80	F	Sporadic	−	Not rec.		
	9	69	F	Sporadic	−	40		
	10	63	F	Sporadic	−	36		
Control	1	59	F	MI	NA	5	MCx and FACx	FFPE and frozen
	2	67	M	HCC		63	MCx and FACx	FFPE and frozen
	3	63	F	Control		Not rec.	MCx and FACx	FFPE and frozen
	4	63	M	CVD		20	MCx and FACx	FFPE and frozen
	5	63	M	Control		Not rec.	MCx	FFPE and frozen
	6	72	M	IHD		31	FACx	FFPE
	7	69	M	Control		Not rec.	FACx	FFPE
	8	78	M	BGC		75	FACx	FFPE
	9	Not rec.	Not rec.	Control		Not rec.	FACx	FFPE
	10	84	M	Age‐related atrophy		Not rec.	FACx	FFPE
	11	54	M	IHD		8	MCx	FFPE
	12	75	M	LB dysphagia		27	MCx	FFPE
	13	53	M	MS		Not rec.	MCx	FFPE
	14	66	F	SMN		Not rec.	MCx	FFPE

ALS/MND, amyotrophic lateral sclerosis/motor neuron disease; BGC, basal ganglia calcification; CVD, cerebrovascular disease; F, female; FACx, frontal association cortex; FFPE, formalin‐fixed paraffin embedded; FTD, frontotemporal dementia; h, hours; HCC, hepatocellular carcinoma; IHD, ischaemic heart disease; LB, Lewy body; M, male; MI, myocardial infarction; MS, multiple sclerosis; MCx, motor cortex; NA, not applicable; Not rec., not recorded; PD, Parkinson's disease; SMN, sensory motor neuropathy; y, years.

### Immunohistochemistry

Immunohistochemistry for p16, p21, γH2AX and 8‐OHdG (8‐hydroxydeoxyguanosine) was performed on FFPE tissue using a standard avidin‐biotin horseradish peroxidase enzyme complex method (Vectastain Universal Elite ABC kit, Vector Laboratories, UK), and the signal was visualized using 3,3′‐diaminobenzidine (DAB) (Vector Laboratories, UK). Briefly, 6‐μm sections were deparaffinized and rehydrated to water. Endogenous peroxidase activity was quenched by incubation of the sections in 0.3% H_2_O_2_/methanol for 20 min at room temperature (RT). After antigen retrieval, sections were incubated in 1.5% normal serum for 30 min at RT, followed by incubation with the respective primary antibody. A summary of the primary antibodies, all commercially available and optimized for this study, and their conditions of use is shown in Table 2. Sections were then incubated with a biotinylated secondary antibody (against specific species depending on primary antibody used) for 30 min at RT, followed by incubation with ABC reagent, for 30 min at RT. To visualize the signal, sections were incubated with the peroxidase substrate solution, then counterstained with haematoxylin, cleared and mounted. Negative controls consisted of sections incubated with omission of the primary antibody and isotype controls.

**Table 2:** Source, specificity, dilution, antigen retrieval and incubation conditions for the antibodies used for immunohistochemistry and double immunofluorescence.

Antibody	Species	Dilution and conditions	Antigen retrieval	Supplier
p16	Mouse monoclonal (IgG)	Prediluted O/N, 4 °C	Pressure cooker, Access Revelation Buffer pH 9.5	BioGenex, UK
p21	Mouse monoclonal (IgG2bκ)	1:100 O/N, 4 °C	MW 10‐min TSC buffer pH 6	Millipore UK Limited, UK
γH2AX	Rabbit polyclonal	1:500 O/N, 4 °C	Pressure cooker, EDTA pH 8	R&D Systems, UK
8‐OHdG	Mouse monoclonal (IgGκ)	1:400 1 hr, RT	Pressure cooker, Access Revelation Buffer pH 6	Abcam, Cambridge UK
NeuN	Rabbit polyclonal (IgG)	1:50 O/N, 4 °C	NA (used for double labelling on frozen tissue)	Proteintech, IL, USA
GFAP	Rabbit polyclonal	1:500 1 h, RT	NA (used for double labelling on frozen tissue)	DakoCytomation, Ely, UK

EDTA, ethylenediaminetetraacetic acid buffer; MW, microwave; NA, not applicable; O/N, overnight; RT, room temperature; TSC, trisodium citrate buffer; 8‐OHdG, 8‐hydroxy‐2′‐deoxyguanosine.

To determine whether glial cells expressing p16 and p21 were astrocytes, double immunofluorescence against p16 or p21 and glial fibrillary acidic protein (GFAP) was performed. For this, 8‐μm frozen sections were fixed in acetone and incubated with 0.2% glycine. Sections were blocked with normal serum (1.5%) for 30 min at RT, followed by an overnight incubation at 4°C with rabbit anti‐GFAP and mouse anti‐p21 or mouse anti‐p16. Sections were then washed and incubated with Alexa Fluor fluorescent secondary antibodies (Thermo Fisher, Waltham, MA, USA) goat anti‐mouse (Alexa Fluor 568, 1:500) and donkey anti‐rabbit (Alexa fluor 488, 1:500) for 1 h at RT. Sections were incubated with Hoechst 33342 solution (Sigma‐Aldrich, St. Louis, MO, USA) to stain the nuclei before mounting with Fluoromount Mounting Media (Sigma‐Aldrich, St. Louis, MO, USA). Double immunofluorescent staining was also performed to confirm the expression of p21 in neurones. For this, acetone‐fixed frozen sections were incubated with rabbit anti‐NeuN (which recognizes nuclear and cytoplasmic NeuN isoforms) and mouse anti‐p21 or mouse anti‐p16 following the protocol described previously. Every run included single‐labelled sections that showed the same staining pattern as seen in the double‐labelled sections. Details of the antibodies used for immunofluorescence experiments are specified in Table 2. Images were captured with a Nikon Eclipse 80i microscope (Nikon UK, Kingston upon Thames, UK), and co‐localization was assessed using the Fiji ImageJ image processing package 24.

### Quantification of p21^+^, p16^+^ and 8‐OHdG^+^ cells

For each case, images from the MCx, FACx and OCx were captured in a belt‐transect pattern, from layer II to the white matter border, as described by Armstrong 25, using a Nikon Eclipse 80i microscope. For p16 and p21 immunohistochemistry, the number of positively stained glial and neuronal nuclei was determined following the protocol used by Al‐Mashhadi *et al*. 26. Briefly, with the help of a grid overlaid on each captured image, glial and neuronal nuclei that were positive to p16 or p21 were counted; the total number of glial and neuronal nuclei was also determined to calculate the percentage of positive nuclei for each marker. Quantitative analysis for p16/p21^+^ glial cells did not differentiate between glial cell types. 8‐OHdG staining was scored following the same protocol, cytoplasmic staining was not assessed as positive, and only clearly visible neuronal nuclei were quantified. All counts were conducted by two independent observers.

### Quantification of γH2AX^+^ cells

Images were captured in a belt‐transect pattern using the 20X objective, excluding layer I, as described in the previous section. Quantification of the total number of γH2AX^+^ nuclei was performed in the MCx and FACx, using Analysis^˄^D software (Olympus Biosystems, Watford, UK). The number of γH2AX^+^ neuronal nuclei was determined using a size exclusion of >10 μm (500 pixels), and the number of positive small nuclei (glia) was determined by subtracting the number of pyramidal neuronal nuclei from the total number of positive nuclei. To assess the total number of cells (neurones and glia), the same detection protocol was applied to haematoxylin‐only stained sections, which allowed the determination of the percentage of immunopositive cells (total number of γH2AX^+^ neurones*100/total number of neurones; total number of γH2AX^+^ glia*100/total number of glia per subject).

### Statistical analysis

Statistical analyses were performed using IBM SPSS Statistics v22. Comparisons between ALS/MND and control groups were done using Mann–Whitney U‐test nonparametric analysis. The effect size was calculated using the formula *r *= Z/√N, where *r* represents the effect size, as proposed by Fritz, Morris and Richler 27, 28; Z represents the Mann–Whitney U‐test Z‐score; and N represents the sample size. Cohen's guidelines for *r* were used for interpretation of the data 27, 28. Associations were determined using Spearman's correlation coefficient (*r*_*s*_).

## Results

Markers of senescence and oxidative DNA damage were examined in the MCx and FACx areas of ALS/MND and control donors. The MCx is an area that is directly affected in ALS/MND and has been studied extensively, whereas the FACx, although not directly implicated in the disease, may show some degeneration that could potentially contribute to the cognitive impairment seen in some ALS/MND patients. The OCx was included as a control area with no pathology, even at late stages. Amyotrophic lateral sclerosis/motor neurone disease and control groups within the cohort did not differ in average age (*P = 0.695*), and there was no difference in mean PMD (*P = 0.613*). None of the markers here assessed correlated significantly with age or PMD (Table S1). Results for the quantitative analysis of the expression of senescence, oxidative stress and DNA damage markers are shown in Table 3.

**Table 3:** Expression of senescence and DNA damage markers in neurones and glia in the ALS/MND and control cohort

Descriptives	Group	Senescence markers	FACx	MCx	OCx	DNA damage/oxidative stress markers	FACx	MCx
Mean (SD) Median (IQR)	Control	p16, glial	12.18 (6.10) 12.92 (11.55)	20.43 (21.35) 13.46 (37.58)	12.03 (3.28) 12.88 (5.94)	γH2AX, neuronal	22.74 (14.57) 24.27 (26.65)	36.66 (22.81) 37.58 (44.06)
	ALS/MND		27.73 (13.81) 24.05 (14.69)	14.46 (11.08) 10.97 (15.99)	14.01 (4.92) 13.51 (6.63)		25.00 (9.91) 24.00 (13.84)	34.65 (30.49) 17.78 (51.34)
	Control	p21, glial	32.63 (4.63) 32.15 (8.68)	25.93 (14.64) 28.80 (27.48)	53.82 (11.37) 49.85 (19.63)	γH2AX, glial	40.28 (28.59) 32.64 (50.38)	56.76 (27.25) 61.39 (50.15)
	ALS/MND		50.46 (10.66) 47.70 (16.60)	35.23 (14.96) 34.45 (27.75)	62.35 (17.32) 67.00 (33.98)		38.97 (13.07) 42.87 (20.64)	48.22 (33.93) 34.85 (61.36)
	Control	p21, neuronal	8.88 (7.87) 8.25 (14.92)	12.40 (10.78) 12.50 (19.40)	56.18 (33.53) 69.35 (56.08)	8‐OHdG, neuronal	78.91 (5.61) 81.50 (8.91)	56.08 (31.20) 55.85 (57.52)
	ALS/MND		32.14 (16.56) 35.20 (21.73)	25.72 (18.88) 27.70 (34.85)	61.20 (35.22) 59.30 (55.75)		85.79 (7.91) 87.93 (13.80)	53.52 (24.61) 54.31 (43.59)

Data are expressed as percentage of positive cells in the brain regions under study.

FACx, frontal association cortex; IQR, interquartile range; MCx, motor cortex; MND, motor neuron disease; SD, standard deviation; 8‐OHdG, 8‐hydroxy‐2′‐deoxyguanosine.

### p16 is expressed by astrocytes, while p21 is associated with both neurones and astrocytes

**Figure 1:**
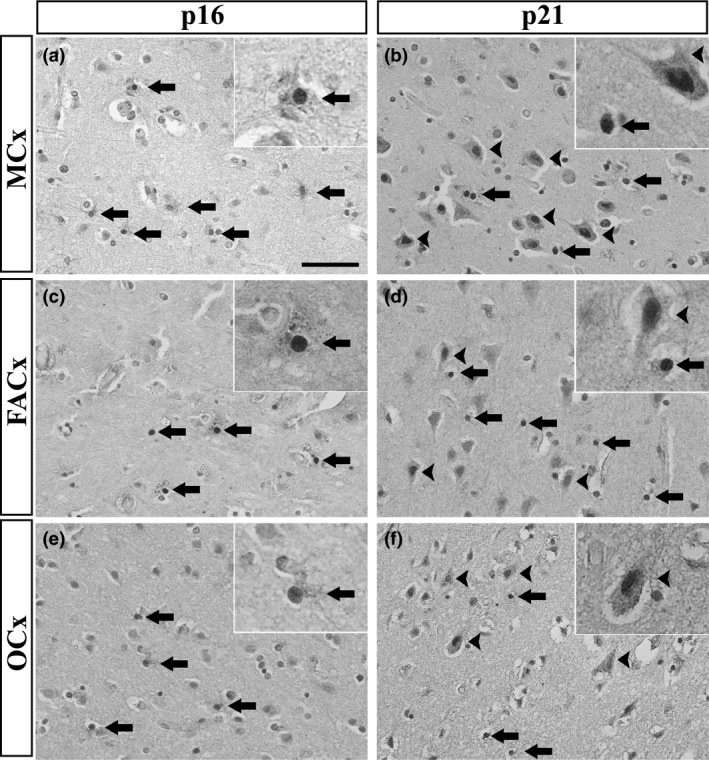
Detection of senescence markers in the MCx, FACx and OCx of ALS/MND donors. p16 immunoreactivity was exclusively associated with glial cells, more likely reactive astrocytes (arrows) (**A**): MCx, (**C**): FACx, (**E**): OCx. p21 immunoreactivity was detected in the nuclei of neurones (arrowheads) and glial cells (arrows) (**B**): MCx, (**D**): FACx, (**F**): OCx. Scale bar represents 50 μm. ALS/MND, Amyotrophic lateral sclerosis/motor neurone disease; FACx, frontal association cortex; MCx, motor cortex; OCx, occipital cortex.

**Figure 2:**
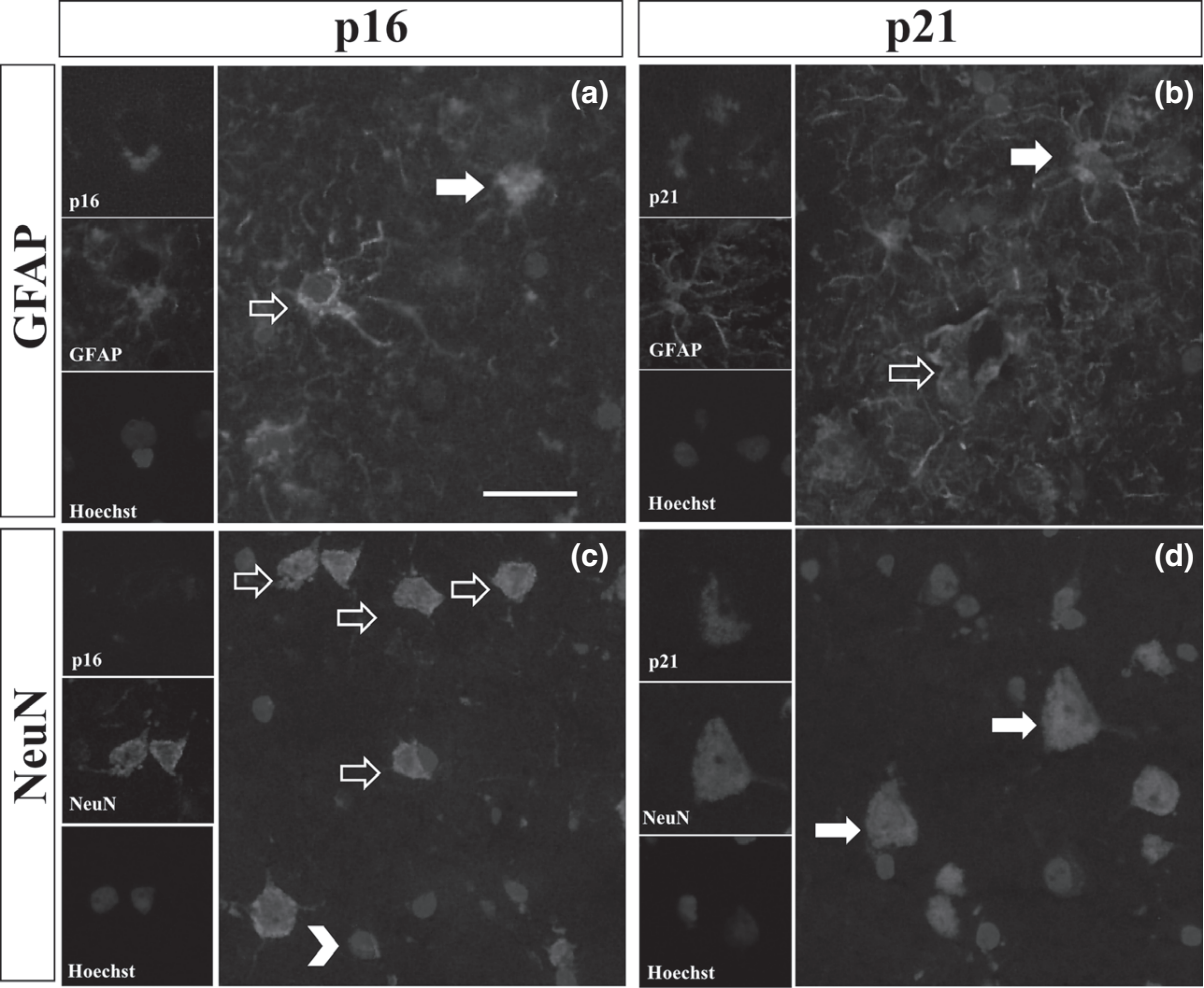
Representative images of double immunofluorescence for p16 and p21 with GFAP or NeuN in the FACx of ALS/MND donors. (**A**) Glial p16 (red) co‐localized with processes of GFAP ^+^ astrocytes (green) (arrows), but not all GFAP expressing astrocytes were positively labelled for p16 (arrow outlines). (**B**) Glial p21 (red) co‐localized with GFAP ^+^ astrocytes (green) (arrows); there was also a population of GFAP ^+^ astrocytes that did not express p21 (arrow outlines). (**C**) Expression of p16 (red) was only localized to glial cells (arrowheads) but not to neurones (arrow outlines). (**D**). Co‐localization of p21 and NeuN confirmed expression of p21 in neurones (arrows). Scale bar represents 25 μm. ALS/MND, Amyotrophic lateral sclerosis/motor neurone disease; GFAP, glial fibrillary acidic protein; FACx, frontal association cortex.

Expression of p16 was detected in the nucleus and cytoplasm of glial cells but not in neurones. Cytoplasmic staining was mainly localized to star‐shaped small cells, which resembled reactive astrocytes and their processes (Figure 1). Double immunofluorescence revealed co‐localization of p16 and GFAP in some cells, confirming them as astrocytes; however, not all GFAP^+^ astrocytes were positive for p16 and vice versa (Figure 2). Small nuclei that did not co‐localize with GFAP immunoreactivity but were positive for p16 could also account for other glial cell types (microglia and oligodendrocytes). Double immunofluorescence of p16 and NeuN, a common neuronal marker, confirmed that p16 was exclusively expressed in glial cells, since p16/NeuN co‐localization was not detected (Figure 2). p16 expression was not only found in ALS/MND brains, but control cases also showed some level of p16 glial staining in the grey matter of both MCx and FACx sections (Figure S1).

Immunohistochemistry against p21 revealed expression of this senescence marker in neurones and glial cells. p21^+^ cells (neurones and glia) were identified throughout the grey matter in the MCx and FACx of ALS/MND (Figure 1) and control donors (Figure S1). Glial and neuronal staining was localized to the nucleus and cytoplasm of cells. p21 cytoplasmic staining in glia localized to the processes of astrocytes and confirmed by co‐localization of p21 staining with GFAP^+^ cells, although as observed for p16, not all GFAP^+^ cells were p21^+^ and there was a population of p21^+^ cells that did not co‐localize with GFAP staining (Figure 2). Double labelling for p21 and NeuN confirmed the expression of p21 in pyramidal neurones of the MCx and FACx of control and ALS/MND donors (Figure 2).

Analysis of the OCx revealed the expression of p16 in glial cells and the expression of p21 in glia and neurones in both ALS/MND (Figure 1) and control cases, as seen in the FACx and MCx (Figure S1).

### The frontal association cortex of ALS/MND donors contains a significantly higher proportion of p16^+^ and p21^+^ cells

**Figure 3:**
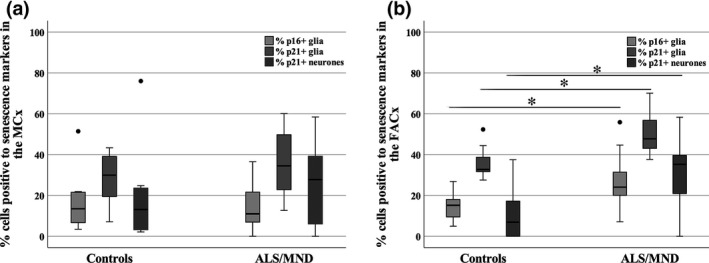
Box plots showing the percentage of p16/p21^+^ cells in the MCx (**A**) and FACx (**B**) of ALS/MND donors versus controls. The percentage of p16/p21^+^ glia and p21^+^ neurones was significantly higher in the FACx of ALS/MND donors, but no significant difference was found in the MCx (*P ≤ 0.05). Controls, *n* = 9; ALS/MND,* n* = 10. ALS/MND, Amyotrophic lateral sclerosis/motor neurone disease; FACx, frontal association cortex; MCx, motor cortex.

Having identified expression of senescence biomarkers in astrocytes and neurones in our cohort, we then quantified the number of cells expressing these markers to establish whether there was a difference between ALS/MND and control donors. For this, the percentages of p21^+^ neurones and p16^+^/p21^+^ glial cells were determined. Quantitation of p16^+^ glial cells resulted in a significantly higher percentage of p16^+^ glial cells in the FACx of ALS/MND donors compared with controls (*P = 0.028*) (Figure 3), but no difference was detected in the MCx (*P = 0.965*) (Figure 3). To further evaluate this difference, we estimated the effect size as described previously 27, 29. The resulting *r* value was used to determine the size of the effect based on Cohen's guidelines for *r* 28, which suggested a large effect size (*r = 0.505*) of the difference in the percentage of p16^+^ glia in the FACx between groups.

Quantification of p21^+^ staining was conducted for glia and neurones. A significantly higher percentage of p21^+^ glia (*P = 0.003*) and p21^+^ neurones (*P = 0.008*) was again found in the FACx of ALS/MND donors when compared to controls, but no difference was detected in the MCx (glia, *P = 0.515*; neurones, *P = 0.360*) (Figure 3). The resulting *r* value suggested a large effect size (*r = 0.656*) of the difference in the percentage of p21^+^ glia in the FACx between groups and a large effect size (*r = 0.605*) in the percentage of p21^+^ neurones in the FACx between ALS/MND and control donors.

Quantification of p16^+^ glial cells and p21^+^ glia/neurones in the OCx did not reveal a significant difference in the percentage p16^+^ glia (*P = 0.968*), p21^+^ glia (*P = 0.780*) or p21^+^ neurones (*P = 0.661*) in ALS/MND cases when compared to controls.

### No association between DDR and markers of senescence in either neurones or glia of ALS/MND patients

**Figure 4:**
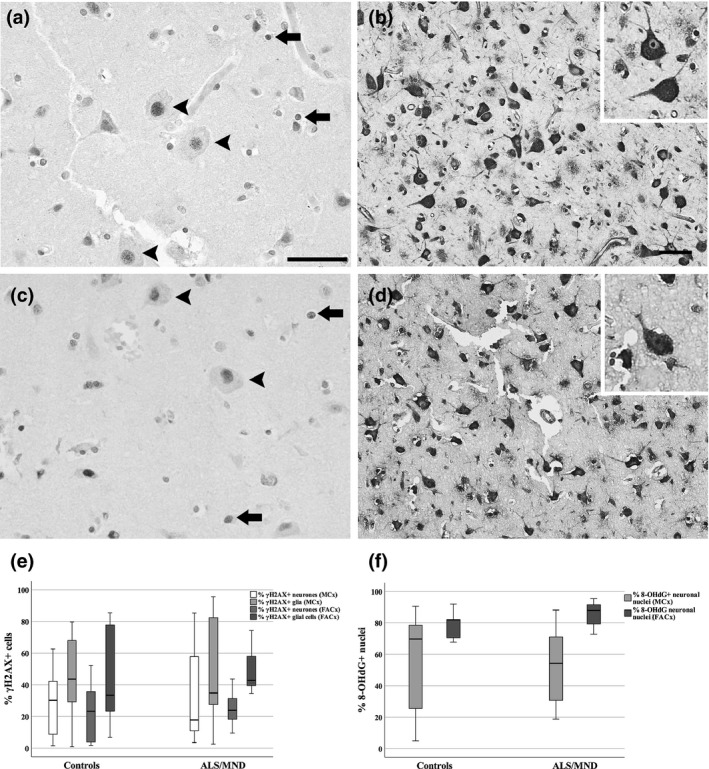
Immunohistochemistry to *γ*H2AX, a DNA damage‐related molecule and 8‐OHdG, a marker of oxidative stress. Nuclear expression of γH2AX was detected in neurones and glia in the MCx (A) and FACx (**C**) of ALS/MND donors, but no significant difference was found in the proportion of γH2AX+ neurones when compared to controls (**E**). 8‐OHdG was detected in the cytoplasm and nuclei of neurones and glial cells (MCx, **B**; FACx, **D**) of ALS/MND cases. No significant difference was detected in the proportion of 8‐OHdG+ neurones in ALS/MND donors when compared to controls (**F**). Scale bars represent 50 μm. Controls, *n* = 9; ALS/MND,* n* = 10. ALS/MND, Amyotrophic lateral sclerosis/motor neurone disease; FACx, frontal association cortex; MCx, motor cortex; 8‐OHdG, 8‐hydroxy‐2′‐deoxyguanosine.

Senescence can be triggered by different stimuli, including oxidative DNA damage and a persistent DDR. To assess whether increased expression of p16 and p21 in the FACx region of ALS/MND donors associated with oxidative stress and a DDR, we used 8‐OHdG and γH2AX to identify oxidative DNA damage and DDR activation. γH2AX immunohistochemical detection revealed nuclear expression in neurones and glial cells (Figure 4). The presence of γH2AX^+^ cells was localized in both ALS/MND and control donors, in the MCx and FACx (Figure 4). Quantification of γH2AX^+^ glia in the MCx and FACx did not show a significant difference in the percentage of positive glia between ALS/MND and control cases (MCx, *P = 0.905*; FACx, *P = 0.661*). Moreover, quantification of γH2AX^+^ showed no difference in the percentage of positive neurones in ALS/MND cases either (MCx, *P = 0.720*; FACx, *P = 0.604*) (Figure 4). Immunoreactivity for 8‐OHdG was detected in the nuclei and cytoplasm of neurones and glia (Figure 4). Due to the variability of the staining, only nuclear immunoreactivity in neurones was assessed by two independent observers. A significant positive correlation between both counts was detected (*r*_*S*_ *= 0.659*,* P ≤ 0.001*), and for further analysis, the mean of the two observers’ measures was used. The percentage of 8‐OHdG^+^ nuclei of pyramidal neurones in ALS/MND and control donors did not differ significantly in the MCx (*P = 0.968*) and in the FACx (*P = 0.243*) (Figure 4).

**Figure 5:**
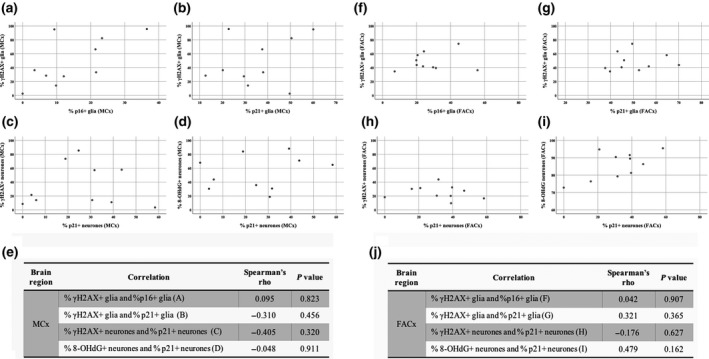
Association between DNA damage and senescence markers in neurones and glial cells in ALS/MND brains. Scatterplots showing no significant correlation between the percentages of γH2AX+ and p16/p21+ glia in the MCx (**A**,** B**) and FACx (**F**,** G**). p21+ neurones did not associate with γH2AX+ or 8‐OHdG expression either (MCx: **C** and **D**; FACx: **H** and **I**). Correlation coefficients and *P* values are summarized in **E** and **J**. ALS/MND,* n* = 10. ALS/MND, Amyotrophic lateral sclerosis/motor neurone disease; FACx, frontal association cortex; MCx, motor cortex.

To determine the relationship between the expression of p16 and p21 senescence biomarkers and the 8‐OHdG and γH2AX in neurones and glia, a statistical dependence analysis between these variables was conducted. The association analysis between the percentages of p21/p16 and γH2AX^+^ glia did not show a significant correlation in the MCx or the FACx of ALS/MND donors (Figure 5). Moreover, the expression of nuclear p21 in neurones did not correlate with γH2AX nor with 8‐OHdG expression in the MCx and FACx of ALS/MND donors (Figure 5).

## Discussion

Increasing evidence has shown accumulation of senescent cells in ageing tissue that could contribute to the development of age‐related pathologies, such as atherosclerosis, osteoarthritis and lung‐disease 30, 31, 32. Senescence in the brain and its relationship to neurodegeneration is less well understood, although there is evidence of expression of senescence markers and the development of a secretory phenotype in astrocytes, microglia and post‐mitotic neurones in the ageing brain 13, 17, 18. These data suggest that induction of senescence in the brain could be an alternative mechanism participating in the development of neurodegenerative diseases. Moreover, the findings of a senescent‐like phenotype in mice and human post‐mitotic neurones indicate that this mechanism might not be exclusive to proliferating cells. In this study, we investigated the presence of senescent cells in the brain of ALS/MND and control donors, and the relationship of this mechanism to the DDR. We found expression of p21 in neurones and glia, whereas p16 was exclusively expressed by glial cells, in both control cases and ALS/MND donors. Double immunofluorescence experiments confirmed that some of the glial cells expressing p16 or p21 were GFAP^+^ astrocytes. These results may suggest an age‐related neuronal p16/p21 up‐regulation linked to cell cycle dysregulation and induction of astrocyte senescence. There was a significantly higher percentage of p16^+^ and p21^+^ glial cells and p21^+^ neurones in the FACx of ALS/MND patients, which could be indicative of these mechanisms contributing to the progression of disease at early stages and their involvement in the cognitive decline observed in a population of ALS/MND patients. Finally, there was no difference in the expression of DNA damage markers in ALS/MND when compared to control donors and correlation analyses did not show significant associations between DNA damage and expression of senescence biomarkers in glia or neurones.

Neurones accumulate DNA damage with age and they are especially vulnerable to DNA insults, due to their high metabolic rate and their limited capacity for cell replacement 19, 33, 34. An increase in ROS, a reduced capacity of antioxidant mechanisms and accumulation of DNA damage are important players in the development and progression of neurodegenerative diseases, including ALS/MND 26, 35, 36, 37. Senescence can be triggered by oxidative stress, DNA damage and a persistent DDR 8, 38, which raises the question of whether these conditions could potentially induce the activation of senescence pathways in brain cells, including post‐mitotic neurones. No specific marker of senescence is yet available, so detection of senescent cells relies on the identification of a set of features that characterize cells undergoing this mechanism. These include up‐regulation of p16 and p21 proteins, as well as DNA damage accumulation and expression of DNA damage response mediators that are indicative of a persistent DDR 39, 40. Using immunohistochemistry against p16 and p21, we show expression of both cell cycle regulatory proteins in glial cells, mainly astrocytes, and of p21 in neurones, in ALS/MND and control brains.

Our results show that neuronal expression of p21 was a common feature in the MCx, FACx and OCx of both control and ALS/MND donors. The expression of this cell cycle regulatory protein in post‐mitotic neurones is not well characterized, but despite their post‐mitotic state, neurones expressing p21 have been linked to a senescent‐like state in a murine model of ageing, where the proportion of p21^+^ neurones increased with the age of mice, together with IL‐6 and DNA damage, proposing induction of senescence as a result of DNA damage accumulation in ageing individuals 18. We identified p21^+^ neurones in ALS/MND brains, which may indicate activation of this pathway and induction of a senescent phenotype; however, p21^+^ neurones were also a feature of control donors in all brain areas, including the OCx, suggesting increased expression of p21 may more generally reflect age‐related cell cycle dysregulation. Several reports indicate that p21 has an essential role in neuronal differentiation and in cell cycle regulation during DNA damage response and repair mechanisms 41, 42. It has been shown that cell cycle re‐entry is necessary for neurones to repair DNA damage or to activate apoptosis 43, 44, 45 and p21 has been linked to the maintenance of neurones in G0 phase under these circumstances, preventing aberrant cell cycle activation and cell death 45. Expression of nuclear p21 in ALS/MND and control donors could be in part related to a normal response to age‐related DNA damage that requires p21 up‐regulation to prevent aberrant S‐phase transition and apoptosis. On the other hand, a recent study reporting defective ATM signalling in spinal cord motor neurones of C9orf72^+^ ALS patients suggests an important role for impaired DDR mechanisms in ALS/MND 46. DDR dysfunction in C9orf72^+^ ALS patients could be linked to defects in DDR‐related neuronal cell cycle re‐entry and could promote aberrant expression of cell cycle regulatory proteins, such as p21. Our data cannot confirm this hypothesis, and further analysis on the cell cycle regulatory pathway in neurones should be undertaken in a larger cohort of ALS/MND and control individuals.

A role for astrocyte senescence in brain ageing has been proposed previously 47, and expression of p16 in astrocytes in our cohort correlates with reports that show increased number of p16^+^ astrocytes in post‐mortem tissue of ageing individuals 13. Moreover, p16 astrocytic expression has been linked to brain degeneration. When compared to age‐matched controls, a significantly higher number of p16^+^ astrocytes was present in the brain of individuals with AD 13, 48 and nuclear expression of p16 was observed in white matter lesions and cortex in ageing brain 19, 26. A recent analysis of p16 and p21 expression in post‐mortem tissue from ALS/MND donors also showed increased number of p16^+^ cells in ALS/MND when compared to controls as well as up‐regulation of p21 mRNA in these cases 48. Our quantitative data showing a significantly higher percentage of p16^+^ and p21^+^ glial cells in the FACx of ALS/MND donors support the proposed involvement of glial senescence in neurodegeneration. It is known that the p16 and the p21 pathways have a different role in the activation and maintenance of a senescent state in mitotic cells. A study conducted in human lung fibroblasts found that p21 is required for the G1‐cell cycle arrest that characterizes senescence in response to DNA damage, while p16 accumulation is necessary for the long‐term maintenance of a senescent state 49. However, the interplay between p16 and p21 pathways is more complicated than described here. For instance, it has been suggested that p16 is not necessary for the induction of telomere shortening‐dependent senescence in human fibroblasts but is rather dependent on the ATM–p53–p21 axis 50. In a different study, a p21‐dependent cell cycle arrest was induced in normal fibroblasts exposed to ionizing radiation and replicative senescence; this pathway was affected in p53‐deficient Li‐Fraumeni syndrome fibroblasts, which instead activated senescence through the p16 pathway 51. Together, this evidence suggests that, apart from participating at different stages of senescence activation, the p16 or p21 senescence‐mediated pathways are triggered depending on the type of stress that the cells are exposed to. Expression of p16 and p21 cell cycle regulatory proteins in astrocytes of ALS/MND and control brains could reflect a pool of cells at different stages of the senescent programme, a pool of cells that have entered senescence as a consequence of different types of stress, or both.

Quantification of p16/p21^+^ glia and p21^+^ neurones revealed a significantly higher percentage of positive cells only in the FACx of ALS/MND cases when compared to controls, but not in the MCx. The FACx (Brodmann area 8/9) is an area that can develop neuroglial inclusions in ALS/MND but at lower prevalence than motor areas, reflected in recent pathology staging approaches 52. The FACx in this ALS/MND cohort can be considered to represent an earlier pathogenetic phase compared with motor areas, providing a pseudo‐time sequence (analogous to the use of Braak neurofibrillary tangle stages for Alzheimer's neuropathology). Moreover, proteomic studies have shown dysregulation of DNA damage and cell cycle regulatory pathways in FTD cases 53. Thus, the significantly higher expression of p21^+^ neurones and p16/p21^+^ glia in the FACx of ALS/MND donors could reflect DNA damage‐related cell cycle dysregulation and a glial senescent‐like mechanism in early stages of ALS/MND.

The expression of 8‐OHdG, a biomarker of oxidative stress in neurodegeneration, confirms that oxidative DNA damage is a prominent feature of both control and ALS/MND cases, where it localizes to the nuclei and cytoplasm of pyramidal neurones, small neurones and glial cells, suggesting the oxidation of nuclear DNA, mitochondrial DNA and RNA. Several studies have identified increased oxidative stress in ALS/MND, which contributes to the pathology of the disease 54, 55, 56, 57, 58. The high proportion of 8‐OHdG^+^ neurones in control donors may be attributed to the accumulation of ROS due to ageing and other disease processes. It remains possible that the degree of neuronal DNA oxidation could be different between ALS/MND cases and controls, but the immunohistochemical detection of 8‐OHdG provides only limited, nonlinear, quantification.

As DNA oxidation induces a DDR, levels of γH2AX were also investigated. Phosphorylation of the histone variant H2AX at Ser139 is an important event in the initiation of the DNA repair response, and since it correlates well with the formation of double‐strand breaks, it has been widely used as a DNA damage biomarker *in vitro* and *in vivo* 59, 60, 61, 62, 63. No difference in the expression of γH2AX was detected in ALS/MND compared with control donors, and the pattern of variation in the expression of γH2AX was more pronounced in the MCx of ALS/MND patients but not in the FACx, where high levels were detected in controls. Analysis of the relationship between senescence and DNA damage markers revealed no significant correlation between 8‐OHdG^+^/γH2AX^+^ and p21^+^ neurones or p16^+^/p21^+^ glia in ALS/MND.

Human post‐mortem tissue studies have a number of limitations, including intrinsic interindividual variability and post‐mortem factors, such as PMD. We did not find a significant association between the levels of senescence and DNA damage markers and the PMD of our cases. The relatively small cohort size could have had an effect on our results by masking differences between groups for levels of DNA damage and senescence. Additionally, our ALS/MND group was not homogeneous; it included sporadic and familial cases, some of which were diagnosed as C9orf72 positive. This could have impacted our results, but when looking at the DNA damage and senescence marker expression in relation to these two variables, we did not find a significant effect within the ALS/MND group. Finally, we did not conduct an analysis of DNA damage and senescence in relation to cognition since information on the cognitive profile of the ALS/MND group was not available. In order to address these limitations, a much larger study of the relationship of DNA damage and senescence to cognitive decline in ALS/MND patients and to specific ALS/MND genetic subtypes would be of value.

In conclusion, we show that glial cells, mainly astrocytes, express p16 and p21, both biomarkers of cellular senescence, in ALS/MND and control brains; neurones, on the other hand, only expressed p21. This could suggest activation of senescence pathways in astrocytes and altered cell cycle in neurones, both mechanisms linked to ageing and the DDR. Moreover, the significantly higher percentage of p16‐ and p21‐positive cells in the FACx of ALS/MND donors could suggest an early involvement of senescence and cell cycle dysregulation in the progression of the disease. Finally, since heterogeneity of the cohort could have affected the study of DNA damage and its relation to senescence markers, further analysis of these mechanisms could be performed in sporadic and familial ALS/MND cases separately, against controls.

## Ethical approval

FFPE and frozen tissue samples were obtained from the Sheffield Brain Tissue Bank (SBTB) following Research Ethical Committee Approval (REC ref 08/MRE00/103).

## Authors contributions

The study was conceived by SBW and JES. IVV planned and carried out the immunohistochemistry experiments and wrote the first draft of the paper. IVV and JES performed the quantitative analyses, and IVV and SBW carried out the statistical analysis. CJG, PRH and PGI provided advice and guidance. All of the authors contributed to supervision of the project and to the final version of the manuscript.

## Declaration of interest

There are no conflicts of interest to disclose.

## Supporting information


**Figure S1.** Detection of senescence markers in the MCx, FACx and OCx of control donors. p16 immunoreactivity was exclusively associated with glial cells, more likely reactive astrocytes (arrows) (**A**: MCx, **C**: FACx, **E**: OCx). p21 immunoreactivity was detected in neurones (arrowheads) and glial cells (arrows) (**B**: MCx, **D**: FACx, **F**: OCx). Scale bar represents 50 μm.

**Table S1**. DNA damage and senescence markers in neurones and glia did not correlate with age or PMD. Summary of correlation coefficients and *p* values obtained from the correlation analysis between DNA damage/senescence markers and age/PMD. Abbreviations: FACx, frontal association cortex; MCx, motor cortex; OCx, occipital cortex; PMD, post‐mortem delay; 8‐OHdG, 8‐hydroxy‐2′‐deoxyguanosine.
