# Supplementary material for: Expression of p16 and p21 in the frontal association cortex of ALS/MND brains suggests neuronal cell cycle dysregulation and astrocyte senescence in early stages of the disease
Source: Neuropathol Appl Neurobiol. 2019 Jun 17;46(2):171–85. doi: 10.1111/nan.12559 (PMC7217199; doi:10.1111/nan.12559)
Supplement: Supplementary file 2 — Table S1. DNA damage and senescence markers in neurones and glia did not correlate with age or PMD. Summary of correlation coefficients and p values obtained from the correlation analysis between DNA damage/senescence markers and age/PMD. Abbreviations: FACx, frontal association cortex; MCx, motor cortex; OCx, occipital cortex; PMD, post‐mortem delay; 8‐OHdG, 8‐hydroxy‐2′‐deoxyguanosine. [file NAN-46-171-s002.docx]

| Correlation with age | | | | | Correlation with PMD | | | | |
| --- | --- | --- | --- | --- | --- | --- | --- | --- | --- |
| Marker | **Brain region** | **Cell type** | **Spearman’s rho (*r_S_*)** | **p value** | **Marker** | **Brain region** | **Cell type** | **Spearman’s rho (*r_S_*)** | **p value** |
| p16 | MCx | Glia | -0.111 | 0.671 | **p16** | MCx | Glia | 0.176 | 0.565 |
|  | FACx |  | 0.498 | 0.062 |  | FACx |  | 0.022 | 0.943 |
|  | OCx |  | 0.431 | 0.074 |  | OCx |  | 0.605 | 0.128 |
| p21 | MCx | Glia | -0.469 | 0.058 | **p21** | MCx | Glia | 0.325 | 0.279 |
|  |  | Neurones | -0.095 | 0.717 |  |  | Neurones | 0.154 | 0.615 |
|  | FACx | Glia | -0.229 | 0.377 |  | FACx | Glia | -0.030 | 0.922 |
|  |  | Neurones | -0.458 | 0.064 |  |  | Neurones | -0.045 | 0.885 |
|  | OCx | Glia | -0.377 | 0.123 |  | OCx | Glia | 0.129 | 0.674 |
|  |  | Neurones | -0.375 | 0.126 |  |  | Neurones | 0.215 | 0.481 |
| γH2AX | MCx | Glia | -0.192 | 0.445 | **γH2AX** | MCx | Glia | -0.176 | 0.565 |
|  |  | Neurones | -0.187 | 0.458 |  |  | Neurones | -0.184 | 0.547 |
|  | FACx | Glia | 0.169 | 0.518 |  | FACx | Glia | 0.195 | 0.523 |
|  |  | Neurones | 0.016 | 0.951 |  |  | Neurones | 0.151 | 0.622 |
| 8-OHdG | MCx | Neurones | 0.340 | 0.168 | **8-OHdG** | MCx | Neurones | -0.179 | 0.559 |
|  | FACx | Neurones | -0.390 | 0.122 |  | FACx | Neurones | 0.138 | 0.654 |
